# Recent Progress in FD-LC-MS/MS Proteomics Method

**DOI:** 10.3389/fchem.2021.640336

**Published:** 2021-06-04

**Authors:** Hiroshi Kobayashi, Kazuhiro Imai

**Affiliations:** ^1^ Laboratory of Proteomics Analysis, Research Institute of Pharmaceutical Sciences, Musashino University, Tokyo, Japan; ^2^ R&D group, Shinwa Chemical Industries, Ltd., Kyoto, Japan

**Keywords:** bio-analytical chemistry, FD-LC-MS/MS method, differential proteomics analysis, spiderweb chromatogram, nano-flow LC, monolithic silica-based capillary column, nano-LC-FD-LC-mass spectrometry

## Abstract

Through the course of our bio-analytical chemistry studies, we developed a novel proteomics analysis method, FD-LC-MS/MS (fluorogenic derivatization-liquid chromatography-tandem mass spectrometry). This method consists of fluorogenic derivatization (FD), LC separation, and detection/quantification of the derivatized proteins, followed by isolation, tryptic digestion of the isolated proteins, and final identification of the isolated proteins using electrospray ionization nano-LC-MS/MS of the generated peptide mixtures with a probability-based protein identification algorithm. In this review, we will present various examples where this method has been used successfully to identify expressed proteins in individual human cells. FD-LC-MS/MS is also suitable for differential proteomics analysis. Here, two biological samples are treated by the same steps mentioned above, and the two chromatograms obtained are compared to identify peaks with different intensities (variation in protein levels). Associated peak fractions are then isolated, and the differentially expressed proteins between the two samples are identified by LC-MS/MS. Several biomarkers for cancers have been identified by FD-LC-MS/MS. For more efficient separation, nano-flow LC with a phenyl-bonded monolithic silica-based capillary column was adopted for cell-expressed intact protein analysis. The derivatized human cell proteins (K562) and yeast cell (*Saccharomyces cerevisiae*) proteins as model intact cell proteins were analyzed by nano-flow LC with fluorescence detection. More than 1,300 protein peaks were separated/detected from both cells. For straightforward comparison of multiple peak separation profiles, a novel type of chromatogram display, termed the “spiderweb” chromatogram, was developed. A nano-LC-FD-LC-mass spectrometry trial for molecular weight estimation of FD proteins has also been conducted.

## Introduction

We will first introduce bio-analytical chemistry (Imai, 1998), which is used by researchers in fields including pharmacology, agriculture, medicine, and biotechnology. Conventional analytical chemists typically develop analytical methods that solve particular problems; however, further application of these methods does not often reach their full potential in fields such as life sciences because the possibilities of these methods are not fully realized or explored. Bio-analytical chemistry is a research field that develops or modifies analytical methods that enable the accurate detection, quantitation, and functional characterization of biomolecules. Thus, as this new field is equally related to biochemistry and analytical chemistry, we have used the term “bio-analytical chemistry” and not “bioanalytical chemistry” to name this discipline.

In bio-analytical chemistry, various types of biomolecules can be examined, including nucleic acids, proteins, amino acids, sugars, lipids, vitamins, hormones, chemical neurotransmitters, and inorganic components. According to this concept, homeostasis is maintained in living mammals, involving blood pressure, body temperature, the pH of the blood, and blood glucose and calcium levels, which are biological markers that can be examined in detail by bio-analytical chemistry.

We will first introduce a bio-analytical chemistry study that examined the maintenance of blood pressure homeostasis. The blood pressure of mammals is primarily controlled by the sympathetic nervous system. A decrease in blood pressure is physically sensed by baroreceptors, which transmit this change to the sympathetic nervous system. Norepinephrine (NE) is then released from sympathetic nerve endings. NE then travels to and is taken up by muscle cells where this hormone/neurotransmitter stimulates muscle contraction. This contraction process causes an increase in blood pressure, and this change is relayed back to baroreceptors to suppress the excitement of the sympathetic nervous system, thereby terminating further muscle contractions. Thus, our blood pressure is constantly regulated. Excess NE can enter the bloodstream and circulate throughout the body, leading to a potential unwanted increase in blood pressure. However, pharmacologists suggested that excess NE is metabolized to inactive normetanephrine (NMN) by catechol-O-methyl transferase (COMT) present in muscle cells. To provide evidence that NE is metabolized to NMN, bio-analytical chemists set about developing a sensitive method to detect and quantitate the extremely low levels (i.e., 10^−12^ mol/l) of NE and NMN simultaneously. Detection using HPLC-chemiluminescence was considered an ideal approach to measure the very low levels of these compounds (Higashidate and Imai., 1992; Prados et al., 1994; Tsunoda et al., 1999; Takezawa et al., 2000a; Takezawa et al., 2000b; Tsunoda et al., 2000; Tsunoda et al., 2001a; Tsunoda and Imai, 2005). Investigating blood pressure homeostasis under normal conditions is challenging because of the small fluctuations in blood pressure and the very low amounts of NE released. Therefore, to expand the range of stationarity, this regulatory system was artificially biased in our investigation (Higashidate et al., 1994; Tsunoda et al., 2000). Here, the blood pressure of rats was lowered by administering a hypotensive agent. Consequently, the baroreceptors received and transmitted that change in blood pressure to the sympathetic nervous system. The sympathetic nerves were excited and released NE from the nerve endings, which acted on the walls of blood vessels to constrict blood vessels, thereby increasing blood pressure to restore normal blood pressure in the animal (Higashidate et al., 1994; Imai et al., 1994). During examination, blood pressure and blood NE and NMN were analyzed. As we hypothesized, the analysis revealed a linear relationship between the decrease in blood pressure and the increase in blood NE levels. Furthermore, in hypertensive rats, this relationship was found to have a steeper slope when compared with that of normal rats, indicating that hypertensive rats have poor sympathetic function (Prados et al., 1997; Prados et al., 1998).

Sympathetic nerve function was shown to be impaired because of constant exposure to hypertension in hypertensive rats. Based on the results, excess NE was not sufficiently inactivated by hypertensive rats, which caused an increase in muscle tension and a consequent rise in blood pressure. A smaller increase in NMN levels was observed in hypertensive rats when compared with that of normal rats. These results indicated that the hypersensitive rats were unable to inactivate NE because of lower levels of COMT when compared with those of normal rats. Such constant exposure to NE was likely responsible for the elevated blood pressure of the hypertensive rats (Tsunoda et al., 2001b; Tsunoda et al., 2003).

A drug that stimulates COMT activity should reduce blood pressure in hypertensive rats; however, there are currently no drugs available that stimulate COMT. Instead, a cofactor that supports COMT activity, S-adenosyl-methionine (SAMe), is available. Administration of SAMe was shown to decrease blood NE levels while simultaneously increasing the blood NMN concentration when compared with the levels before injection of SAMe. Thus, SAMe functions as a drug that lowers blood pressure (Imai et al., 1999). Arginine, a common amino acid, was also found to reduce hypertension in rats (Prados et al., 1999).

In summary, bio-analytical chemistry can be used to analyze life-maintaining functions such as body temperature, the pH of blood, and blood glucose and calcium levels by monitoring fluctuations in the levels of various biomolecules including nucleic acids, proteins, amino acids, sugars, lipids, vitamins, hormones, chemical neurotransmitters, and inorganic components.

## Fluorogenic Derivatization-Liquid Chromatography-Tandem Mass Spectrometry Proteomics Method (FD-LC-MS/MS)

In the Introduction, we described an example in which the mechanism of homeostasis at the individual level was analyzed by bio-analytical chemistry. In this section, methods developed to characterize proteins and their biofunctions at the tissue and cellular level are presented. Note, however, that transmission of information among tissues is lost when individual cells are removed from animals or humans.

**FIGURE 1:**
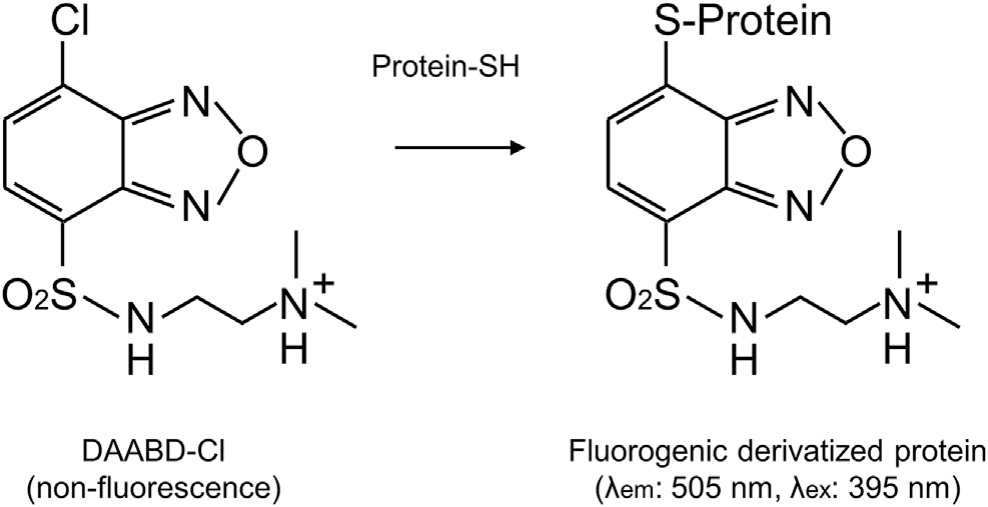
Derivatization of proteins with the fluorogenic reagent DAABD-Cl.

Recently, we have synthesized a new fluorogenic reagent, DAABD-Cl {7-chloro-N-[2-(dimethylamino)ethyl]-2,1,3-benzoxadiazole-4-sulfonamide}, which does not fluoresce but produces fluorescent-labeled proteins, i.e., DAABD-proteins (detection at 505 nm, excitation at 395 nm), after reaction (fluorogenic derivatization) with cysteine residues of the proteins (Miseta and Csutora, 2000) (Figure 1). Background fluorescence is low even when high amounts of DAABD-Cl are used.

We have also developed a method for proteomics analysis (FD-LC-MS/MS) (Toriumi and Imai, 2003; Masuda et al., 2004; Koshiyama et al., 2013a) using DAABD-Cl as a fluorogenic reagent and separation of DAABD-proteins by HPLC. The FD-LC-MS/MS method consists of fluorogenic derivatization (FD) of proteins (FD proteins) using DAABD-Cl, followed by highly reproducible HPLC separation, quantification and isolation of the DAABD-proteins, enzymatic digestion of the isolated proteins, and final identification of the proteins using nano-LC and tandem MS with a database search algorithm. In a conventional FD-LC system, the detection limit of FD proteins and peptides ranges between 0.5 and 30 fmol (Imai, 2014). The proposed method has similar sensitivity to electrophoretic and conventional LC-MS/MS methods but is superior in terms of simplicity and by way of identification of proteins. The specifications of each proteomics method (Iwasaki et al., 2010; Zhang et al., 2013; Imai, 2014; Nakata et al., 2015; Blundon et al., 2019; Lee et al., 2019; Kobayashi et al., 2021) are summarized in Table 1.

**TABLE 1:** Specifications of each proteomics method.

Method	FD-LC-MS/MS	Two-dimensional differential gel electrophoresis (2D-DIGE)	Shotgun
Separation target	Protein	Protein	Peptide
Quantification target	Protein	Protein	Peptide
Qualification target (MS target)	Protein or peptide	Protein or peptide	Peptide
Detection reproducibility	Fine (fluorescence detector)	Fair (digital image scanner)	Fair (mass spectrometry)
Batch-to-batch repeatability	Fine	Low	Fair
Sample amount (µg)	1–16^a–d^	100–250^e^	1–46^f^ ^,^ ^g^
Protein fractionation	Fraction collector	Manual spot picking	-
Limit of detection (fmol)	0.5–30^a^ (protein)	0.2^e^ (protein)	0.065^g^ (peptide)

a Imai, 2014.

b Nakata et al., 2015.

c Lee et al., 2019.

d Kobayashi et al., 2021.

e Blundon et al., 2019.

f Zhang et al., 2013.

g Iwasaki et al., 2015.

**FIGURE 2:**
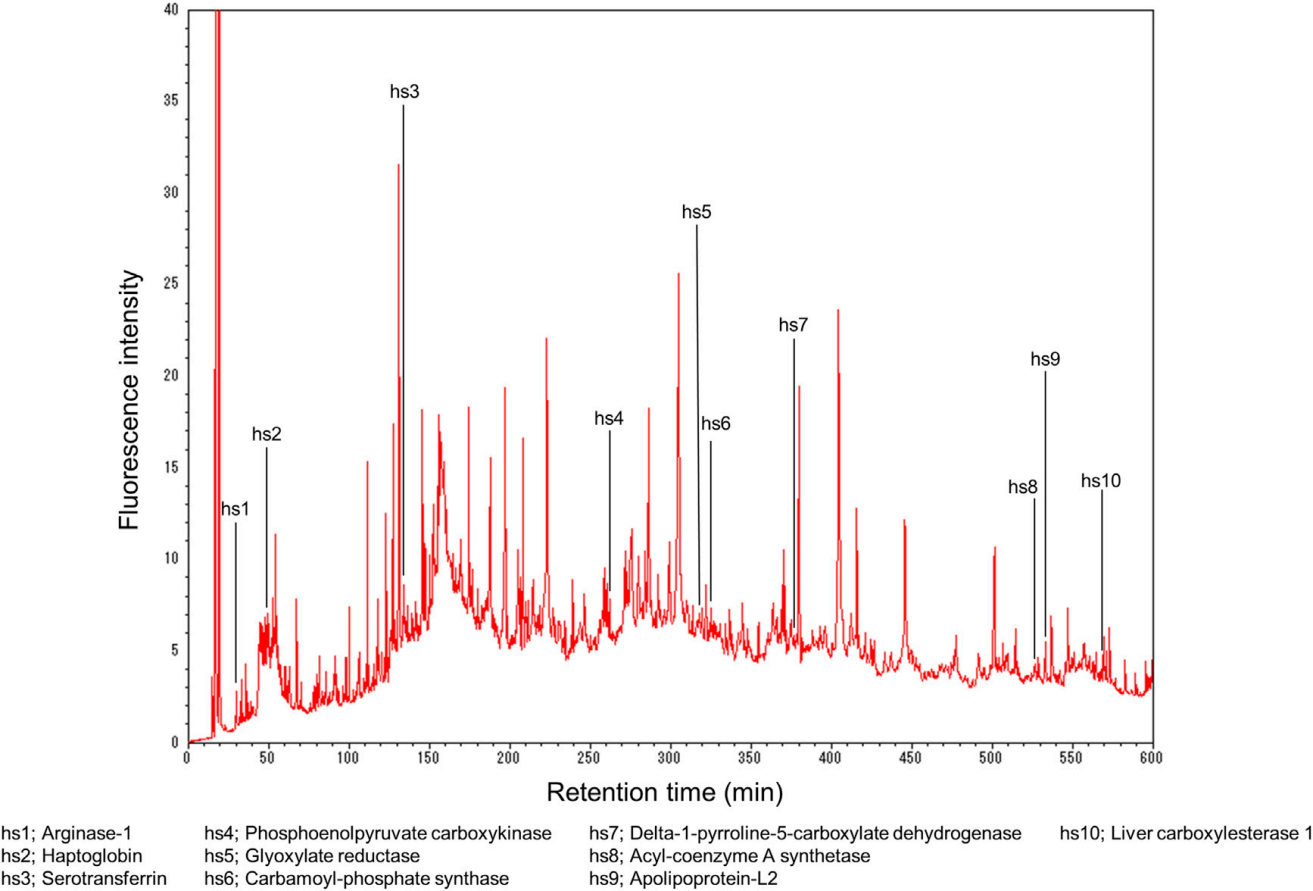
Fingerprint analysis of hepatocyte cell proteins. Reprinted with permission from Nakata et al. (2015).

Figure 2 shows the example of the proteomics analysis of an HepaRG cell (terminally differentiated hepatic cells derived from a human hepatic progenitor cell line) extract (Nakata et al., 2015). The experimental details are described below.

An HepaRG protein lysate (40 µg/35 µl) was mixed with 40 µl of a mixture of 1.25 mmol/l tris(2-carboxyethyl)phosphine hydrochloride (TCEP) and 5.0 mmol/l ethylenediamine-N,N,N′,N′-tetraacetic acid sodium salt (Na_2_EDTA) in guanidine buffer (pH 8.7), 20 µl of 2.5% Triton X-114 in guanidine buffer (pH 8.7), and 5.0 µl of 140 mmol/l DAABD-Cl (around 100-fold molar excess against proteins) in acetonitrile. After the reaction mixture had been placed at 40°C in a water bath for 10 min, 3.0 µl of 20% (v/v) trifluoroacetic acid (TFA) was added to terminate the derivatization reaction. Twenty microliters (equivalent to 8.0 µg protein) of the reaction mixture were injected into the LC system. The three connected columns (750 mm long) used are core–shell materials (WIDEPORE XB-C8, 250 × 4.6 mm i.d., 3.6 µm particle, Phenomenex, Torrance, CA, United States) with a column temperature of 60°C, and the gradient program was as follows: 0→5→20→25→40→120→450→510→585→600 min; B 0→0→30→30→30→40→60→70→100→100% [(A) acetonitrile:isopropanol:water:TFA = 9.0:1.0:90:0.10 (v/v/v/v); (B) acetonitrile:isopropanol:water:TFA = 74:1.0:25:0.10 (v/v/v/v)]. The corresponding flow rate was 0.40→0.40→0.40→0.20→0.20→0.20→0.20→0.20→0.20→0.20 ml/min.

The eluate from the LC was fractionated and digested with trypsin, and the proteins were identified by using a nano-LC-ESI-MS/MS system (UltiMate 3000, LTQ Orbitrap XL, Thermo Fisher Scientific, Waltham, MA, United States). The tryptic digestion of the fractionated proteins was conducted as follows. Each protein eluate was concentrated to 5.0 µl under reduced pressure. The residual material was diluted with 50 µl of 250 mM ammonium bicarbonate solution (pH 7.8) containing 0.50 U trypsin (Promega, Madison, WI, United States) and 10 mM calcium chloride. The reaction mixture was incubated for 2.0 h at 37°C. The solution was subsequently concentrated to 20 µl. The identification of proteins was conducted by using Mascot search engine software for the MS/MS spectrum against the protein database. The variable modification was set as DAABD for cysteine residues because proteins were modified with DAABD-Cl in the FD-LC-MS/MS method.

Five hundred thirty-two peaks were detected, and furthermore, as depicted, 10 hepatocyte-specific proteins (hs1–hs10) were found in the HepaRG extract: hs1, arginase-1; hs2, haptoglobin; hs3, serotransferrin; hs4, phosphoenolpyruvate carboxykinase [GTP]; hs5, glyoxylate reductase/hydroxypyruvate reductase; hs6, carbamoyl-phosphate synthase [ammonia]; hs7, delta-1-pyrroline-5-carboxylate dehydrogenase; hs8, acyl-coenzyme A synthetase ACSM2B; hs9, apolipoprotein-L2; and hs10, liver carboxylesterase 1. Therefore, we termed this approach “proteomics fingerprint analysis of individual cells” because the FD-LC-MS/MS method identified proteins within human liver cells.

### Comprehensive Differential Proteomics Analysis Between Cells and Tissues

**FIGURE 3:**
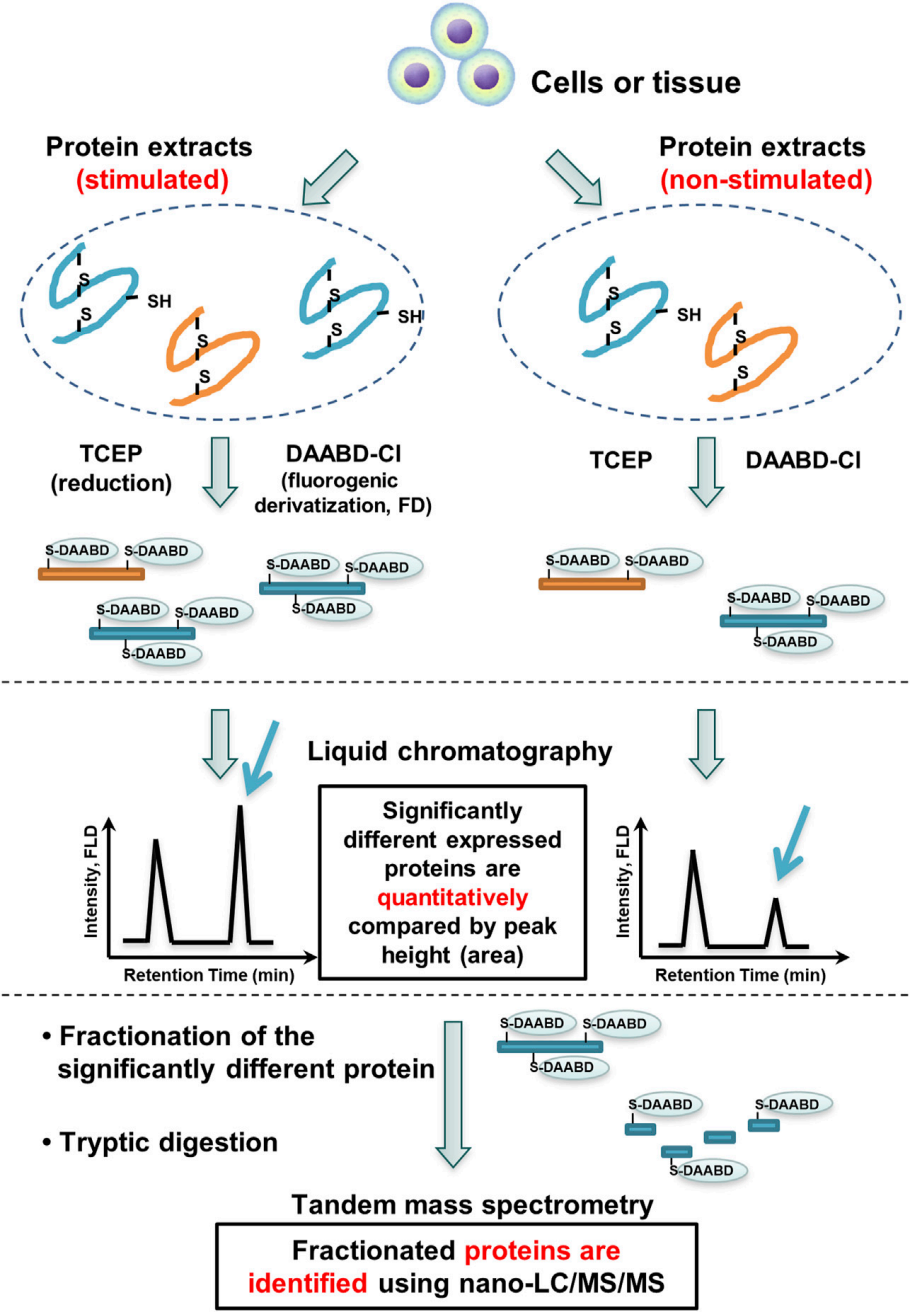
FD-LC-MS/MS method for comprehensive differential proteomics analysis.

The FD-LC-MS/MS method is also suitable for differential proteomics analysis (Figure 3). Two different biological samples are treated in the same manner described above. Proteins in one mixture, A, are derivatized with DAABD-Cl, and proteins in the other mixture, B, are also derivatized in the same manner. The two chromatograms A and B are compared, and the corresponding peak heights are quantified. Corresponding peak heights that differ significantly between the two chromatograms indicate differentially expressed proteins. These identified proteins are isolated and subjected to enzymatic digestion, and the generated peptide mixtures are analyzed by nano-LC-tandem MS with a database search algorithm to identify the differentially expressed proteins. Identification of these proteins provides information about differential expression of particular proteins among cells.

The method has been applied to differential proteomics analysis in real biological samples such as human cancer cell lines (Imai et al., 2008; Koshiyama et al., 2013b), a hepatitis-infected model mouse (Ichibangase et al., 2007), developing mouse brain small regions (Asamoto et al., 2008), horse skeletal muscle tissues (Ichibangase and Imai, 2009), and immunoaffinity-isolated cellular proteins (Nakata et al., 2018). Importantly, because several proteins are identified simultaneously, this method can link several proteins to new signaling pathways in one experiment.

**FIGURE 4:**
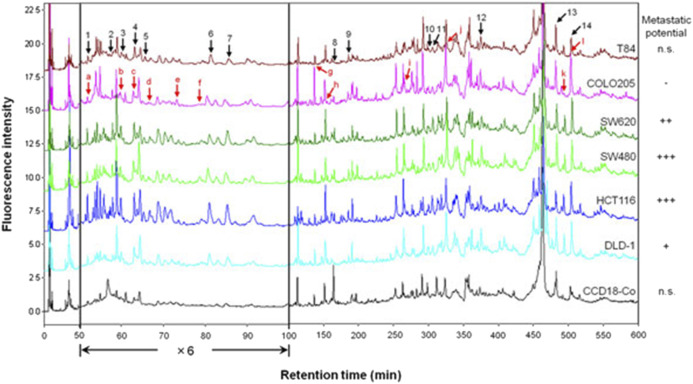
Chromatograms corresponding to 8.0 µg of total protein obtained from six human colorectal cancer cell lines and a normal colorectal cell line after derivatization with DAABD-Cl. The peaks indicated by black arrows (1–14) differed in intensity between the cancer cell lines and the normal cell line. The peaks indicated by red arrows (a–l) differed in intensity between the metastatic cell lines and the non-metastatic cell line.

Figure 4 shows an example performed to find biomarkers by comprehensive differential proteomic analysis of six human colorectal cancer cell lines (T84, COLO205, SW620, SW480, HCT116, and DLD-1) and a normal colorectal cell line (CCD18-Co) (Koshiyama et al., 2013a). The chromatograms correspond to 8.0 µg of total protein obtained from the six human colorectal cancer cell lines and the normal colorectal cell line after derivatization with DAABD-Cl. Fourteen proteins with fluctuating levels of expression were identified, and from these data, we have proposed a model explaining how the dynamic expression of these proteins affects colorectal cancer tumor progression. In a comprehensive differential analysis of four metastatic cell lines (SW620, SW480, HCT116, and DLD-1) vs. the non-metastatic cell line COLO205, 12 differentially expressed proteins were identified, several of which may be secreted and influence the development of metastasis. For further details, refer to Koshiyama et al. (2013b).

We have also analyzed seven breast cancer cell lines, one of which was malignant, MDA-MB-231, and the other six benign (HCC1428, AU565, MDA-MB-468, SKBR-3, MCF7, and BT-474) (Imai et al., 2008). A normal cell line, HMEC, was used for comparison. Several hundred derivatized protein peaks appeared in each chromatogram. After comparison of each peak height, 13 significantly different protein peaks from HMEC proteins were obtained. Highly upregulated proteins in breast cancer cells were identified: ran-specific GTPase-activating protein (RanBP-1) and peroxiredoxin-1. Moreover, several upregulated proteins were also identified, including cofilin-1, Raf-1 kinase inhibitor protein (RKIP), profilin-1, Ras-related nuclear protein (Ran), thioredoxin-1, and Ras homology GDP dissociation inhibitor (RhoGDI). Additionally, we showed that several proteins were downregulated such as stratifin, galectin-1, annexin-2, and elongation factor Tu (EF-Tu). Tropomyosin-1 was expressed solely in normal cells. Some of the expressed proteins, such as RKIP and annexin-2, positively correlated with the estrogen receptor (ER) or human epidermal growth factor type 2 receptor (Her2). The observation of cooperatively expressed annexin-2 and galectin-1 in breast cancer cells without tropomysin-1 is indicative of metastasis. Based on the expression levels of other proteins identified in the breast cancer cell lines, the Rho signal stimulation pathway was deduced to be a key pathway that facilitated proliferation of and disorder within cancer cells. For further details, refer to Imai et al. (2008).

Biomarker discovery for interstitial renal fibrosis caused by aristolochic acid has been reported as another practical application of this method. In the mouse model, changes in the expression level of two proteins, thrombospondin type 1 and G protein–coupled receptor 87, which is a tumorigenesis-related protein, were discovered (Lin et al., 2018). Additionally, the FD-LC-MS/MS method was used to study the mechanism of nephrotoxicity induced by the antimicrobial agent gentamicin (GM). In a rat model, 49 differentially expressed proteins were identified. The most significant mechanisms of nephrotoxicity caused by GM were mitochondrial dysfunction, fatty acid metabolism, and oxidative stress, and their upstream regulator was demonstrated to be peroxisome proliferator–activated receptor alpha (PPARα) (Lee et al., 2019).

### Development of the Nano-LC-FD-LC System

**FIGURE 5:**
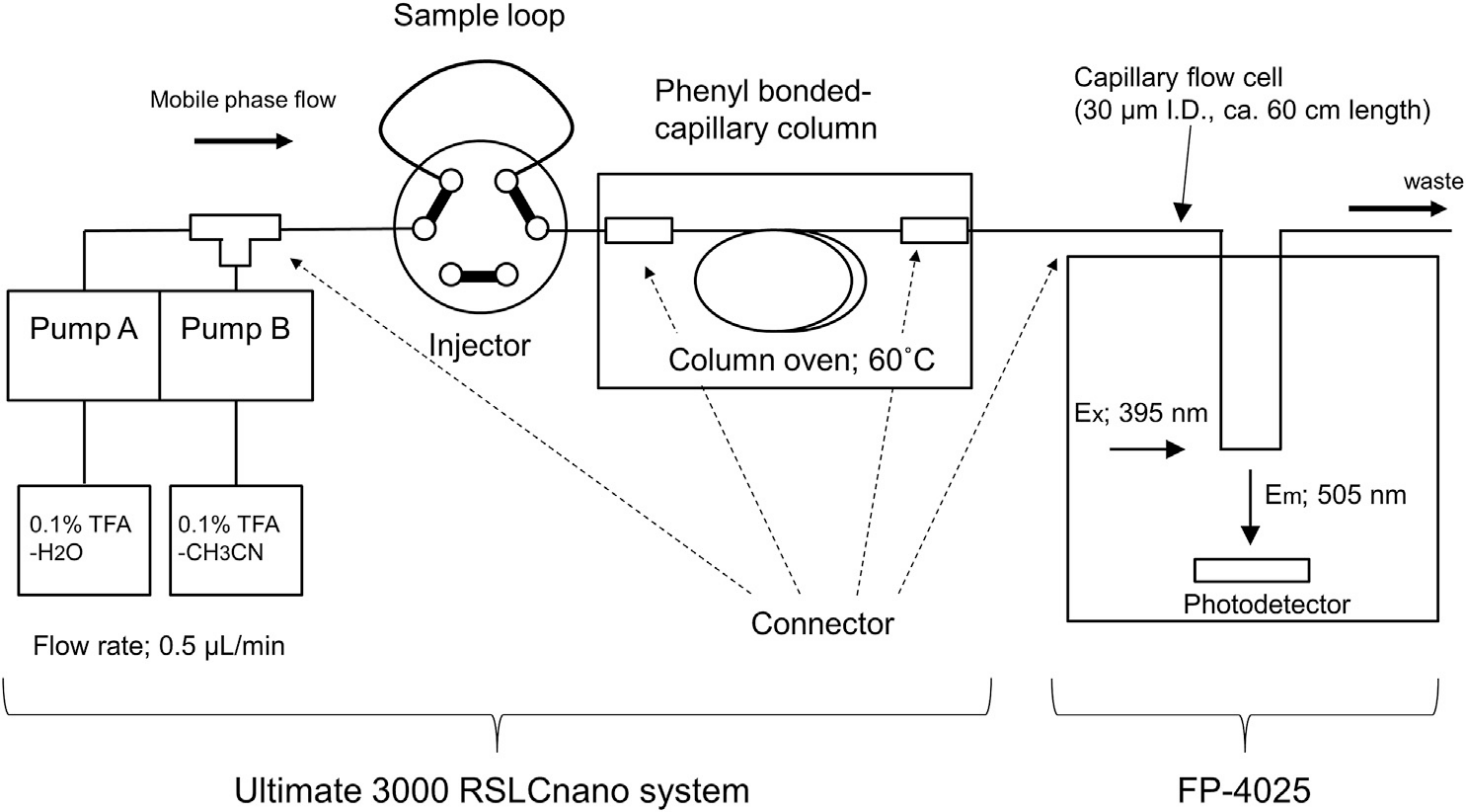
Schematic diagram of the developed nano-flow FD-LC system.

Although the FD-LC-MS/MS method is a valuable approach for comprehensive differential analysis of whole cell–expressed proteins, the performance of this method to discover biomarker proteins is highly dependent on the resolution of the HPLC column. Currently, three 250-mm core–shell coupling columns can resolve approximately 500 protein peaks. From a physiological research perspective, the number of proteins associated with signaling pathways through protein–protein interactions in human cells has been reported to be 8,710 for at least one interaction and 2,015 for five or more interactions (Prasad et al., 2009). Recently, we found that a monolithic silica capillary column modified with phenylsilane was suitable for the separation of intact proteins (Kobayashi et al., 2021). In this study, a 700-mm long phenyl-modified monolithic silica capillary column (internal diameter: 100 µm) was prepared and combined with a nano-LC-FD system to increase the number of proteins that can be resolved by the FD-LC-MS/MS method (Figure 5). A Thermo Scientific Ultimate 3000 RSLCnano system was used as the main nano-LC system. Since fluorescence detectors optimized for nano-LC are not commercially available, the fluorescence detector for this system was a customized commercial LC fluorescence detector (FP-4025, JASCO) that incorporated a capillary tube with an internal diameter of 30 μm and a length of 600 mm as the flow cell.

**FIGURE 6:**
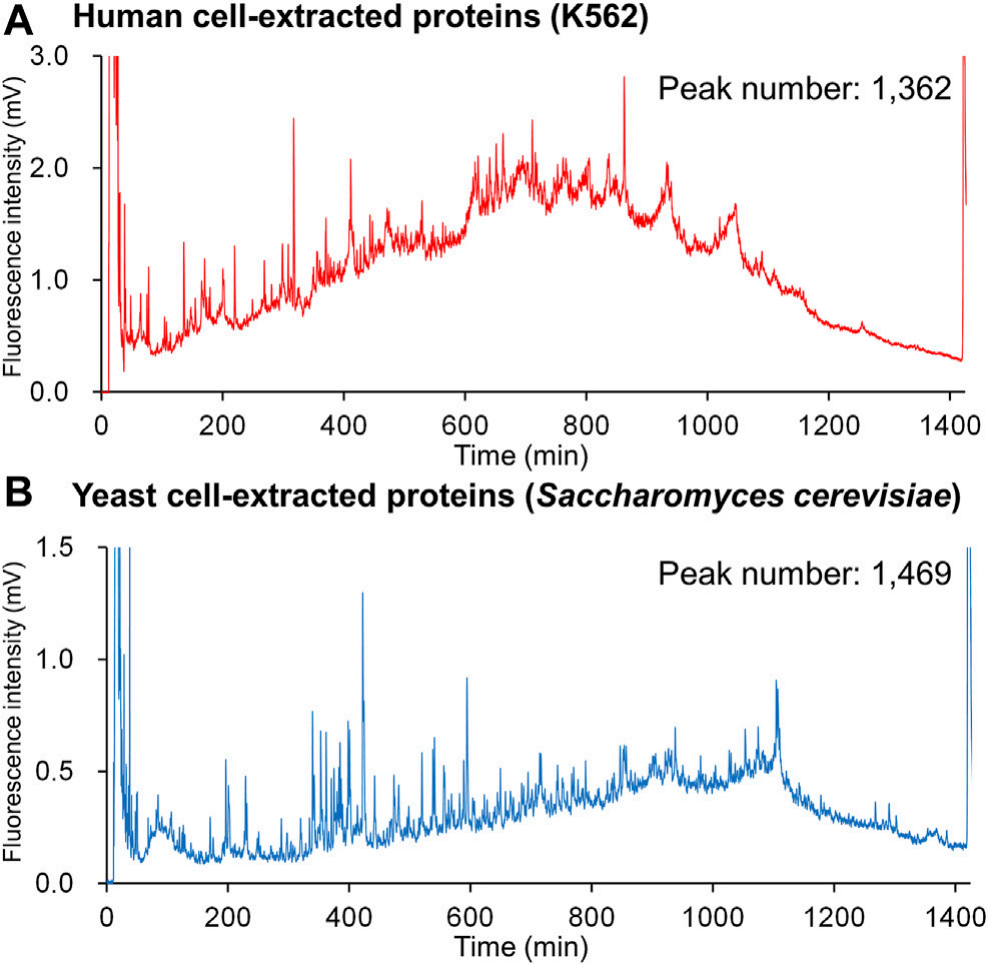
Chromatograms for DAABD-derivatized **(A)** human cell–extracted proteins and **(B)** yeast cell–extracted proteins using the nano-flow FD-LC system.

Figure 6 shows the fluorescence chromatograms obtained from a 1,400 min (ca. 24 h) gradient analysis of DAABD-labeled human (K562) cell–extracted proteins (2 µg) and yeast (*Saccharomyces cerevisiae*) cell–extracted proteins (1 µg) using the developed nano-FD-LC system. The details of the sample preparation and the nano-LC-FD conditions are as follows.

One hundred microliters of human (K562) and yeast (*Saccharomyces cerevisiae*) cell–extracted protein solution [a concentration of 10 µg/µl in 6.5 mol/l urea, 50 mmol/l tris-HCl buffer (pH 8)] were separately dissolved in 6 mol/L guanidine-HCl buffer (pH 8.7) to a total volume of 320 µl. Then, 200 µl each of 50 mmol/l CHAPS in 6 mol/l guanidine-HCl buffer, 10 mmol/l EDTA in 6 mol/l guanidine-HCl buffer, and 2.5 mmol/l TCEP in 6 mol/l guanidine-HCl buffer, and 50 µl of 140 mmol/l DAABD-Cl in acetonitrile were added and mixed. The reaction mixture was incubated at 40°C for 10 min using a thermo-shaker at 800 rpm, and the reaction was stopped with 30 µl 20% TFA_aq_ under ice-cold water conditions. This reaction yielded an FD protein solution with a concentration of 1 mg each/ml (cell-extracted proteins). The nano-LC-FD conditions were as follows: mobile phase A, 0.1% TFA-H_2_O; mobile phase B, 0.1% TFA-CH_3_CN; flow rate, 0.5 µl/min; column, phenyl-modified monolithic silica capillary column (internal diameter: 100 µm; length: 700 mm); column temperature, 60°C; injection volume, 1.0 µl for FD yeast cell proteins and 2.0 µL for human cell proteins; detection, 505 nm with excitation at 395 nm; detector gain, 1,000; and gradient conditions, 1,400 min linear elution of 25%B–45%B and 29%B–49%B for FD yeast cell proteins and FD human cell proteins, respectively.

The number of peaks obtained for each sample was 1,362 for human and 1,469 for yeast, respectively. In a previous study (Nakata et al., 2015) using three connected columns (WIDEPORE XB-C8, 250 × 4.6 mm i.d., 3.6 µm particle, Phenomenex), 532 protein peaks were detected (Figure 2), indicating that nano-flow FD-LC provides superior resolution when compared with that of conventional FD-LC.

Under the same gradient conditions, the range of elution times of the numerous proteins contained in each sample varied, suggesting that different tissues or species have different gradient elution condition requirements. Interestingly, because the elution order of proteins in reversed-phase chromatography is dependent on the organic solvent concentration in the mobile phase (Snyder et al., 2009), the chromatograms in Figure 6 suggest that proteins in human K562 cells may be more hydrophobic than those present in yeast.

As described above, the use of a high-resolution monolithic silica capillary column has improved the separation performance of FD-labeled proteins. However, higher resolution is still required for analysis of expressed proteins in whole cells, and further studies are required to improve the conventional FD-LC-MS/MS method, e.g., use of longer columns and multidimensional LC separation. Furthermore, as with conventional proteomic methods, the dynamic range of proteins is also an issue. For example, in the case of serum analysis, albumin, which exists abundantly, inhibits separation and detection. Therefore, as in standard proteomic approaches (Luque-Garcia and Neubert, 2007), a sample pretreatment method is essential for serum proteomics investigation by FD-LC-MS/MS.

#### Spiderweb Chromatogram

**FIGURE 7:**
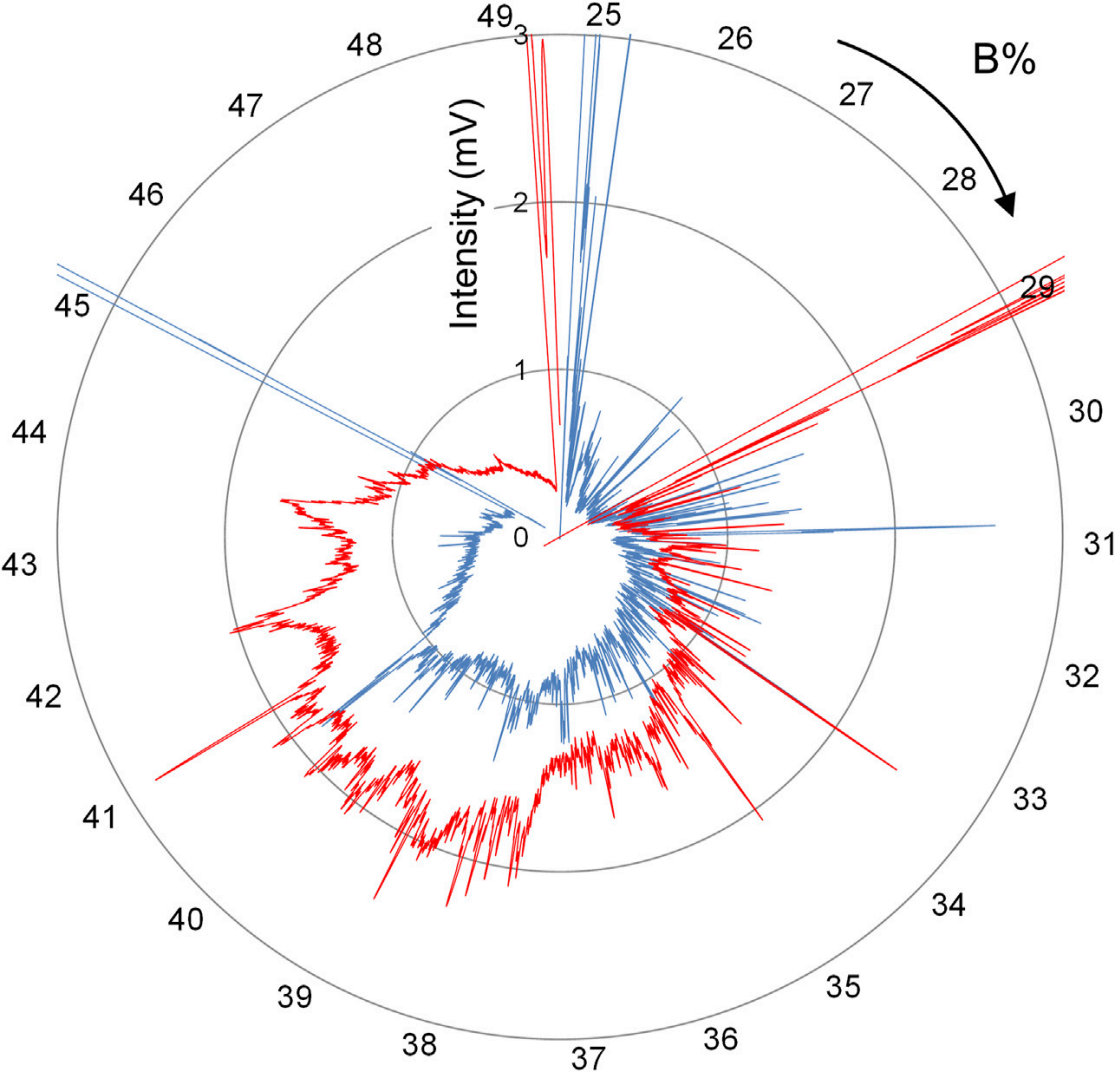
Spiderweb chromatograms obtained by converting chromatograms depicted in Figure 6.

As presented in Figure 6 **,** when the number of peaks obtained in one chromatogram is greater than 1,000, the orthogonal coordinate system typically used in HPLC cannot be used effectively to determine the distance between adjacent target peaks because of their poor separation. To visualize and compare differences among numerous protein peaks obtained by FD-LC, we developed a new chromatogram display system, the spiderweb chromatogram, which displays the chromatogram in the radar chart format (Kobayashi et al., 2021). Figure 7 shows spiderweb chromatograms using the conventional chromatograms presented in Figure 6. In the spiderweb chromatogram, the distance from the center of the circle is used as the detection value and the circumference is used as either the time axis or the percentage of organic solvent concentration in which the protein is eluted. Adjacent peaks are separated at an angle, which increases separation when compared with the standard orthogonal coordinate system. Quantitative visualization of primary data of biologically expressed proteins should facilitate analysis of variations in the expression profiles of proteins, for example, in serum samples before and after drug administration, or changes caused by genome editing.

#### Trial of Nano-LC-FD-LC-MS for Molecular Weight Estimation of FD Proteins

In the conventional FD-LC/MS/MS method, protein peak fractions are identified by LC-MS/MS analysis followed by tryptic digestion. As an alternative, in the present nano-flow FD-LC method, sub-μl scale eluents are adapted by customizing the flow cell of a commercial fluorescence detector. However, the amount of eluate from the column is so small that it is difficult to collect the eluate for the final digestion and identification steps. Thus, rather than identifying separated proteins by peptide mapping, we attempted to measure the molecular weight by connecting the capillary column end directly to MS (Kobayashi et al., 2021). We hypothesized that mass spectrometry should provide molecular weight information about FD proteins because the DAABD molecule has an ionic charge.

**FIGURE 8:**
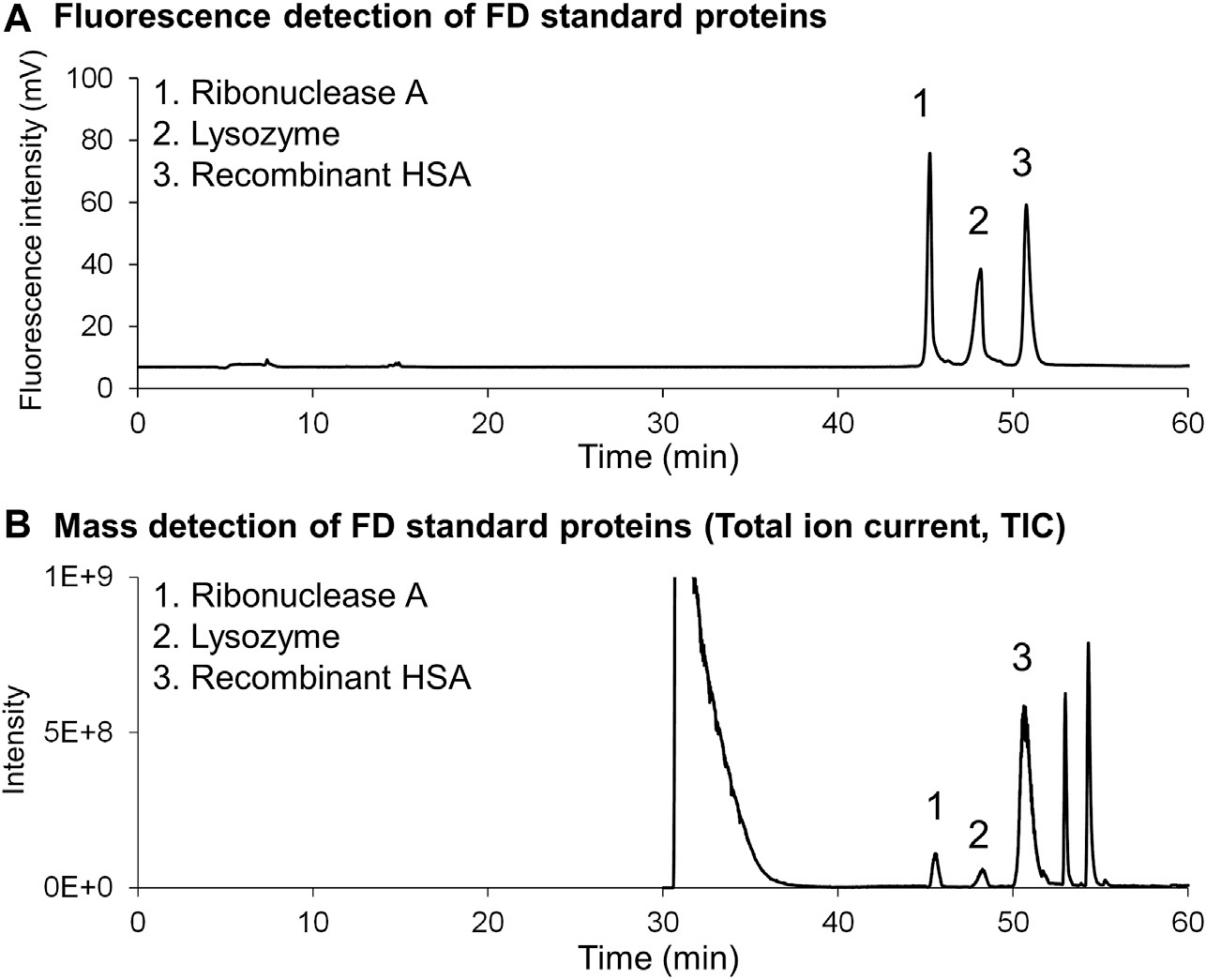
Chromatograms of the **(A)** fluorescence detector and **(B)** mass spectrometer obtained for FD standard proteins dissolved in highly concentrated buffers (6 mol/l guanidine, 10 mmol/l CHAPS, 1 mmol/l EDTA, and 0.5 mmol/l TCEP).

**FIGURE 9:**
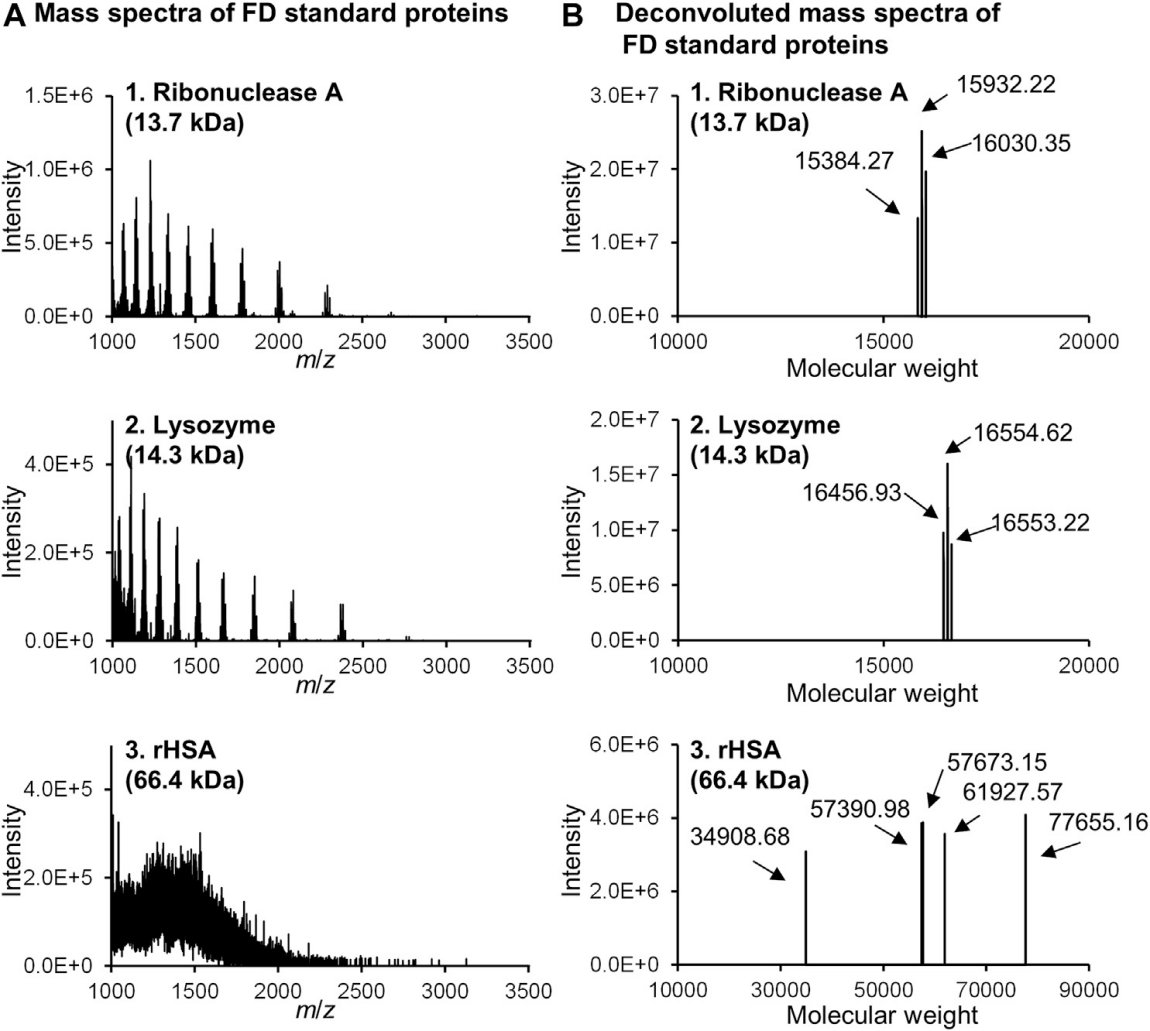
Mass spectrometric analysis of FD standard proteins obtained by using the nano-flow FD-LC/MS system. **(A)** Obtained mass spectra of three FD proteins and **(B)** deconvoluted MS spectra of them.

**FIGURE 10:**
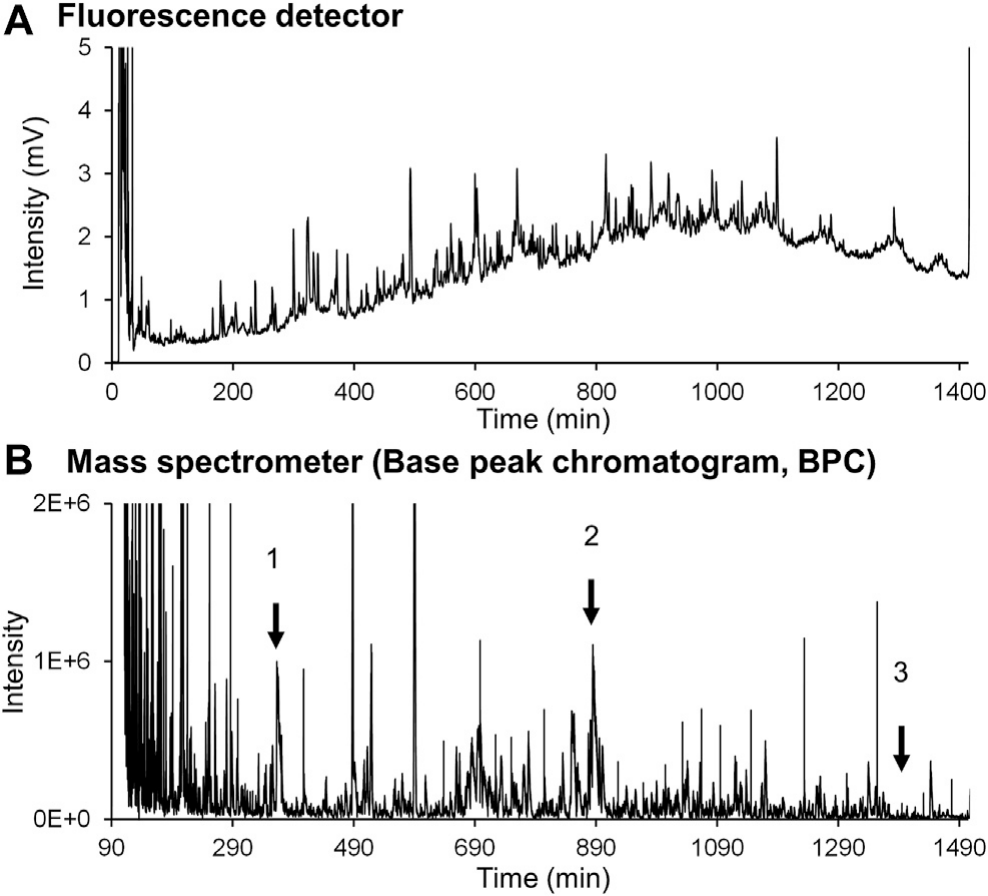
Chromatograms of the **(A)** fluorescence detector and **(B)** mass spectrometer obtained for DAABD-labeled human (K562) cell–extracted proteins over 1,400 min gradient conditions.

Two problems arose when introducing FD protein fractions directly into online MS. The first problem was inhibition of ion detection caused by the high concentration of buffers and surfactants in the column effluent. The second issue was the relatively low detection sensitivity of the proteins, which is usually experienced for peptide analysis. Careful examination revealed that the large amounts of buffers and surfactants in the effluent affected ionization of the FD proteins. Thus, the elution conditions were changed. The first elution was isocratic to remove buffers and surfactants, and then a gradient elution was employed to separate FD proteins on a 250-mm long phenyl monolithic silica capillary column. Three FD proteins (ribonuclease A, lysozyme, and HSA recombinant) were subjected to this LC-MS system to check that the elution conditions are appropriate. The chromatograms obtained are shown in Figure 8A (FD detection) and Figure 8B (MS detection). Figure 9 shows the MS spectra and deconvolution spectra given by each peak in Figure 8B. For the theoretical value of each protein, the measured values are in an appropriate range but are not precise because the MS spectra were not accurate. Unfortunately, we do not have MS/MS available, which provides more precise mass analysis to give accurate deconvolution values. Notwithstanding, we proceeded with real cell sample experiments. Figure 10 shows (A) the fluorescence chromatogram of FD K562 proteins obtained using a 700-mm long phenyl monolithic silica capillary column and (B) the base peak chromatogram (BPC) obtained by MS detection using the same column. For fluorescence detection, gradient elution (22%B–42%B over 1,400 min) started immediately after sample injection, whereas for MS detection, isocratic elution (13%B over 90 min) was performed after sample injection to elute the buffers and other substances, followed by gradient elution (13%B–22%B over 2 min, 22%B–42%B over 1,400 min). The shift in the baseline for fluorescence detection is not caused by the mobile phase solvent but is due to insufficient separation of the FD proteins. Baseline separation will be achieved by further improving separation. Despite the long gradient elution time (about 24 h), stable chromatograms were obtained for both the fluorescence and MS detectors. As seen in Figure 10, the chromatograms obtained with fluorescence detection and MS detection had relatively similar detection patterns. This may be because DAABD-Cl used for protein fluorescence has a tertiary amine as well as a fluorescence emission, which may increase the ionic strength of the protein in MS.

**FIGURE 11:**
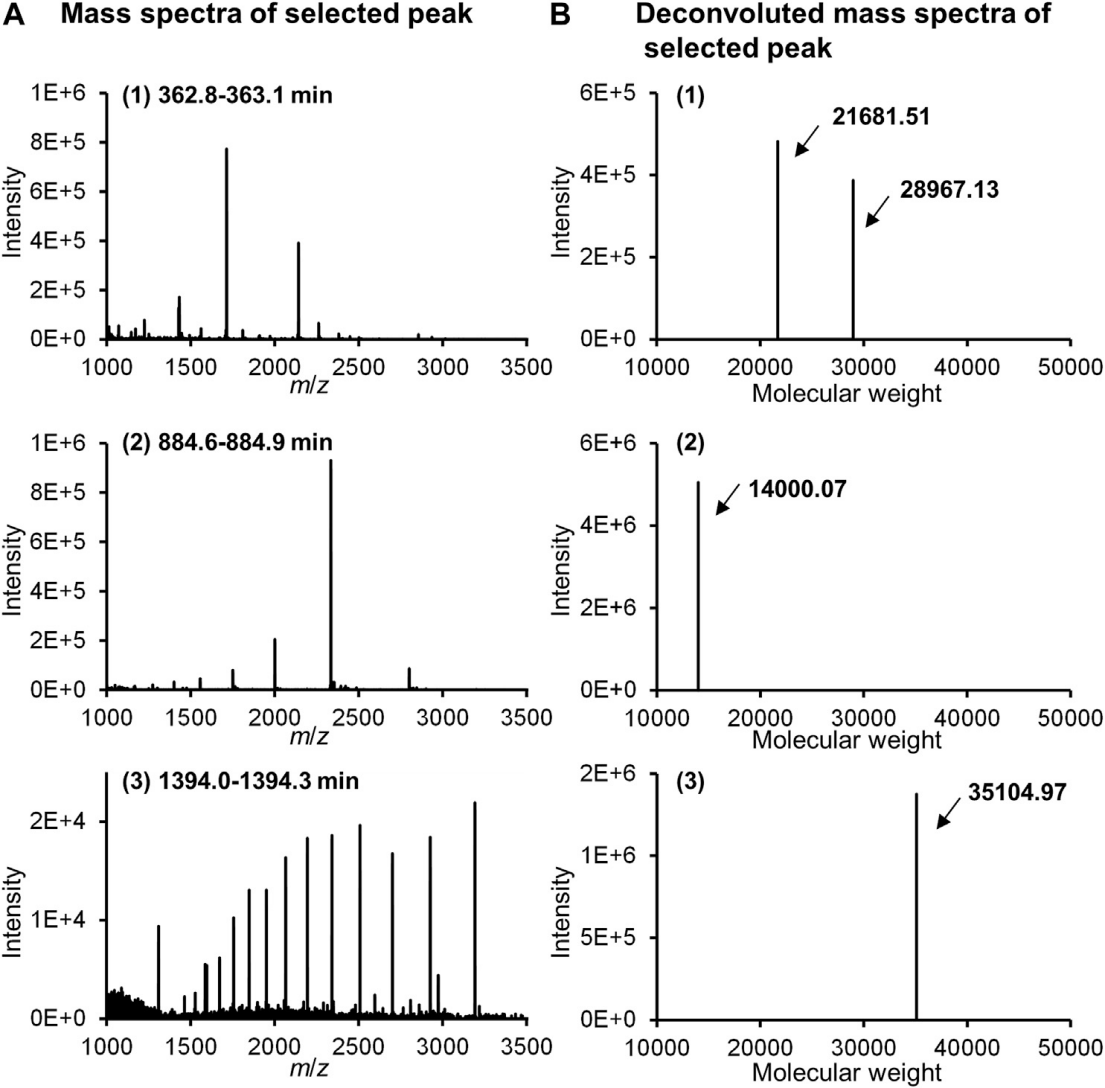
MS spectra **(A)** and deconvoluted MS spectra **(B)** of selected peaks in Figure 10B.

Figure 11 shows the MS spectra of each peak selected in Figure 10B. Thus, stable MS spectra were obtained over the entire long analysis time range of approximately 24 h, suggesting that online nano-flow FD-LC/MS/MS is a feasible method for molecular weight determination of FD proteins in this nano-flow system.

**FIGURE 12:**
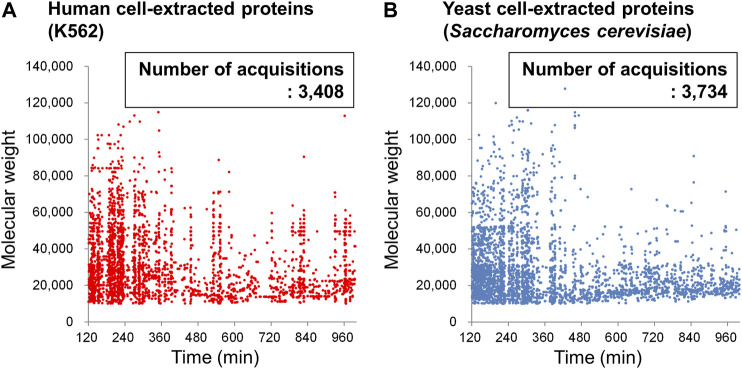
Plots of the deconvoluted molecular weight of DAABD-labeled **(A)** human (K562) cell–extracted proteins and **(B)** yeast cell–extracted proteins against elution time on the nano-flow FD-LC/MS system.

Finally, the results of automatic deconvolution of the obtained BPC for DAABD-derivatized K562 proteins and yeast proteins are shown in Figure 12. Due to software limitations, the deconvolution time range is 120–999 min, which is approximately 2/3 of the gradient range. However, plotting the molecular weights of the detected proteins against the elution time afforded the molecular weight of 3,408 proteins and 3,734 proteins for human K562 cells and yeast cells, respectively. The plot of elution time vs. molecular weight of each protein may be useful for comparative analysis of the expressed proteins in each cell, as well as for 2D gel electrophoresis.

## Future Outlook

The nano-flow FD-LC/MS system we have developed enables quantitative fluorescence detection and qualitative mass spectrometry analysis of whole cell–extracted proteins, suggesting that this system may be suitable for differential cellular characterization. As mentioned above, for identification of proteins in the present nano-flow FD-LC method, we did not identify separated proteins using the commercial fluorescence detector because this detection system is not suitable for the sub-µL scale of the eluent. The potential use of a capillary tube flow cell for the nano-flow LC fluorescence detector should enable collection of the eluent from the flow cell using an automated micro-fraction system. This can then be followed by tryptic digestion and identification of the isolated proteins by LC/MS/MS analysis. The development of this system represents a future challenge. In addition, a tandemly connected fluorescence detector and mass spectrometer would quantify and semi-identify the whole cell–expressed proteins, but only when improvements to mass spectrometry systems and enrichment of databases have been achieved.

Improvement in the separation performance is essential for obtaining more accurate cellular protein expression information. Currently, using a 700-mm long column, detection of approximately 1,400 proteins by fluorescence and 3,700 proteins by MS is possible. Because the number of genetically encoded cysteine-containing proteins in humans is estimated to be 19,871 from the UniProt database (https://www.uniprot.org/, proteome ID: UP000005640), which correspond to ∼97% of human proteins reviewed (20,379), improvement in the performance of the column and LC conditions is required to separate all cell–expressed proteins. Then, this method can integrate and contribute fully to “The Human Protein Atlas, 2020,” project and other key proteomic-based projects.
